# Talent management related concepts and debates in container shipping industry by an emerging market perspective

**DOI:** 10.1186/s41072-021-00090-6

**Published:** 2021-06-17

**Authors:** Ramazan Özkan Yildiz, Soner Esmer

**Affiliations:** 1 Barbaros Hayrettin Naval Architecture and Maritime Faculty, Iskenderun Technical University Iskenderun Technical University (ISTE) Rectorate, Central Campus, 31200 Iskenderun, Hatay Turkey; 2 Maritime Faculty, Dokuz Eylul University, Adatepe Str., Doguş Avenue, Tınaztepe Campus, 35390 Buca, Izmir Turkey

**Keywords:** Talent, Talent management, In-depth interview, Qualitative content analysis, Container shipping industry

## Abstract

Talent management (TM) is referred as a young and developing field and it is claimed to be require more contribution from the different industry and country perspectives. Because of the highly dynamic and competitive nature of the container shipping industry, possession of valuable and unique human capital assets is evaluated as a substantial necessity to achieve business objectives and sustained competitive advantage by container shipping companies. Accordingly, TM is considered to be an essential factor contributing the accomplishment of these goals and the successful implementation of corporate strategy. Therefore, this study aims to contribute the development of TM field by investigating and evaluating TM related concepts and debates in Turkish container shipping industry. Through this extent, a combined method of content analysis with in-depth interview, has been organized to analyse TM oriented subjects in container shipping industry.

## Introduction

Although talent and talent management (TM) concepts have received a lot of attention in academic studies in recent years, many studies have expressed the lack of intellectual and theoretical foundations (Scullion et al., 2016; Marinakou and Giousmpasoglou, 2019, p.3856). The TM field is still considered to be a not fully established field that is still evolving, and researchers often reiterate that there is a need for consensus on definitions of talent and talent management notions. Gallardo-Gallardo and Thunnissen (2016) argue that very few empirical studies have concentrated on the conceptualization of talent and TM concepts. In addition, Nijs et al. (2014) suggest that more studies should be done from different country and industry perspectives on how talent and talent management is conceptualized in order to develop concepts by analyzing and incorporating different views (Collings and Mellahi, 2009; Tansley, 2011; Vaiman et al., 2012; Chung and D’Annunzio-Green, 2018; Marinakou and Giousmpasoglou, 2019). Vaiman et al. (2012) acknowledge that, this will help to counteract an overly ethnocentric or Anglo-Saxon conceptualization of talent management which is not reflective of practice in many parts of the world.

Associatively, Zhang and Bright (2012) assert that, before implementing talent management, in order to create a comprehensive talent management system, there are basic questions that organizations should first investigate. These are; what the talent is, who is considered to be talent and what does talent management mean. Answering these questions will enable organizations to perceive the components of the system they want to establish on the basis of talent and to shape their procedures and processes accordingly (Bagheri et al., 2020, p.88). More specifically, according to Tansley (2011), a working definition of talent and talent management is necessary for sound talent management policies and practices shared in the organization, and structuring these definitions is vital for talent development professionals to be able to design and plan talent oriented training and development programs. However, choosing a talent definition is not an easy task, especially as there are many ways to define talent within a particular organization.

It is frequently emphasized in the literature that talent and talent management concepts are context-dependent, but the current definitions are not created by looking at different perspectives and especially by evaluating the opinions of the practitioners (Al Ariss et al., 2014; Krishnan and Scullion, 2017). Moreover, Schuler et al. (2011) argue that these definitions have mostly been formed in a western perspective and are generally based on the context of developed countries. Dependently, Khoreva and Kostanek (2019) assert that, no study has been detected which examines the concept of talent and TM conceptualization from an employer perspective in emerging markets. It is claimed that not understanding the employer perspective regarding these two important definitions in emerging markets will lead to problems such as the inability to fully construct the talent management components in these markets and the occurrence of errors in the implementation phase (Khilji et al., 2015; Sidani and Al Ariss, 2014). Moreover, to the best of authors’ knowledge, there is no specific study has been encountered which investigates the meaning of the talent and talent management concepts neither by shipping industry nor by Turkish perspectives in the current literature. As stated earlier, determining exactly what talent and talent management mean and investigating the approaches and debates on these concepts will reveal how talent management should be implemented in a specific sector, on which components it should be built, and what should be the focus.

Through this extent, this study primarily aims to investigate the interpretation of the unique, valuable and inimitable human capital (talent) within the container shipping industry, and secondly, it aims to investigate how the differentiated and specialized management of these idiosyncratic assets (talent management), is described in the industry, by the perspectives of HR executives of container shipping companies operating in Turkey. And also it is aimed to investigate, how talent and talent management centred approaches and discussions are handled in the Turkish container shipping industry, whether there is a different perspective from the general understanding and how these concepts dissociate in different types of companies within the sample (e.g. overseas-based vs Turkish). In accordance with these purposes, in the first part a detailed review of the current literature regarding talent, talent management, TM-oriented approaches concepts have been demonstrated, and also, these concepts have been reviewed within the scope of shipping industry and Turkey. Then, in the methodology section a combined method of in-depth interview and qualitative content analysis has been adopted to critically analyse and identify talent and talent management centred concepts within the container shipping industry. This combined method has been guided by following research questions:

- *Research Question 1: How do the HR executives of Turkish container shipping companies understand the concept of talent management?*
- *Research Question 2: To what extent TM in Turkish container shipping companies is similar with global shipping industry?*
- *Research Question 3: What implications may these findings have in TM in emerging economies such as Turkey?*

## Talent management: existing concepts and debates

In the business management literature, there is no unanimous definition of talent exists (Thunnissen et al., 2013a, p.1754; Thunnissen et al., 2013b, p.327; McDonnell et al., 2017, p.109; Golubovskaya et al., 2019, p.4109; Harsch and Festing, 2020, p.45). When firms’ perceptions on the meaning and scope of talent are examined, it is observed that their considerations on this issue are shaped and varied according to their own organizational dynamics, inherently, this situation makes it difficult to develop a de facto definition (Iles et al., 2010, p.180; Tansley, 2011, p.266; Latukha, 2015, p.1061; Chung and D’Annunzio-Green, 2018, p.104; Bagheri et al., 2020, p.88). Yet, there are some important attempts regarding to the characterization of the talent phenomenon (see Table 2 in Appendix 1).

When the concept of talent is examined, four main well-established comparative trend of approaches, utilized to determine its meaning and scope, draw attention. These are: object vs. subject; innate vs. acquired; input vs. output; transferable vs. context-dependent (Dries, 2013, p.272; Meyers and van Woerkom, 2014, p.193; Bolander et al., 2017, p.1525: Cui et al., 2018, p.11; Marinakou and Giousmpasoglou, 2019, p.3858; Kravariti and Johnston, 2020, p.80).

Object vs. subject approach is regarded to the classification between; talent as employees (subject approach) and talent as characteristics of employees such as KSAs (object approach) (Thunnissen et al., 2013b, p.327; Mensah, 2015, p.548; Bolander et al., 2017, p.1525). The subject approach refers: rare, valuable, inimitable and difficult to replace employees, it reflects the basic assumptions of human capital theory (Lepak and Snell, 2002, p.519; Thunnissen et al., 2013b, p.327; Mensah 2015, p.548), and resource based view (Barney 1991, p.102; Dries, 2013, p.279). On the other side, object approach represents qualifications (e.g. knowledge, skill and abilities, competencies, capabilities) of an employee which drives his/her potential, performance and contribution.

Innate vs. acquired approach is related with nature-nurture debate, which contemplates whether talent is inborn (innate approach), or something can be nurtured with adequate learning and training (acquired approach) (Meyers et al., 2013, p.305; Meyers and van Woerkom, 2014, p.194; Bolander et al., 2017, p.1525; Marinakou and Giousmpasoglou, 2019, p.3858; Meyers et al., 2020, p.563). The innate approach, evaluates talent as a stable and fixed entity and identifies it as a natural ability that cannot be taught (Meyers et al., 2013, p.306; Pantouvakis and Karakasnaki, 2018, p.651). According to the acquired approach, talent is a combination of distinct competencies that each employee can accumulate through a sequence of education, training and experiences. This approach handles talent as, something could be developed and managed (Tarique and Schuler, 2010, p.127; Chabault et al., 2012, p.329; Dries, 2013, p.279; Latukha, 2015, p.1062; Golubovskaya et al., 2019, p.4118).

Input vs. output perspective is focuses on whether talent depends on employees’ motivation (input approach) or capability (output approach) (Dries 2013, p.280; Mensah 2015, p.548; Sparrow and Makram, 2015, p.254; Bolander et al., 2017, p.1525). Input approach, identifies talent as the effort, ambition, interest and values of an employee, while, output approach, qualifies it as an employee’s performance, achievement and contribution to the company (Dries, 2013, p.280; Mensah, 2015, p.548; Bolander et al., 2017, p.1525).

Finally, transferable vs. context-dependent view, relies on the discussion, if talent can be seen as a qualification that maintains unaltered during the transfer between contexts, or as a harmony which grows just in specific contexts (Dries, 2013, p.280; Bolander et al., 2017, p.1525). Context-dependent view suggests that talent is conditional on its environment, however, transferable view assumes that talented people can demonstrate their talent regardless of the working environment (Dries, 2013, p.280).

Talent management has emerged as a remedy to the problem of talent shortages in world-wide business environment (Chambers et al., 1998, p.46; Axelrod et al., 2001, p.9). However, there is still no uniform understanding regarding to the definition of talent management in business management literature exists (Lewis and Heckman, 2006, p.139; Collings and Mellahi, 2009, p.304; Iles et al., 2010, p.185; Scullion et al., 2010, p.106; Valverde et al., 2013, p.1833–1834; Al Ariss et al., 2014, p.173; Ewerlin and Süß, 2016, p.144). Efforts to create a general definition of talent management will be futile, because each business creates the scope and meaning of talent according to its own dynamics, and naturally their understanding and approach to talent management also differ. As a result of this fact, trying to form common expressions in an area where subjectivity is so intense can be considered as an almost impossible action (Burbach and Royle, 2010, p.415; McDonnell et al., 2011, p.178; Jones et al., 2012, p.402; Pandita and Ray, 2018, p.187; Bagheri et al., 2020, p.88). Nevertheless, there are important attempts of academic researchers related with the definition of talent management (see Table 3 in Appendix 2), which can be sufficiently suited to the academic need to stimulate theory development while reflecting the interests of practitioners (Garrow and Hirsh, 2008, p.390; Thunnissen et al., 2013a, p.1749–1750; Cappelli and Keller, 2014, p.307; Tafti et al., 2017, p.16).

In the process of reviewing the literature on talent management, two prominent disputes stand out. While the first of these concerns is regarding to the form of the relationship between talent management and human resources management; the second one pays attention to feature and context of the employee group on which talent management should focus (inclusive vs. exclusive approach).

In the discussion on the relationship between talent management and human resources management, one party sees talent management as a part of human resources management and describes it as a garnishing of what currently exists (old wine in the new bottle), not being different from traditional HRM practices or disciplines (Lewis and Heckman, 2006, p.140; Iles et al., 2010, p.180; Al Ariss et al., 2014, p.173; Cooke et al., 2014, p.226; Festing and Schäfer, 2014, p.263). By contrast with that, the other side refers talent management as a differentiated management systematic, by its, focal point (talent in an exclusive and/or inclusive way); integration with all business dynamics (e.g. corporate culture, business objectives, business strategies etc.); design and implementation (e.g. unique, valuable and inimitable functions, strategies and practices); and contribution (e.g. enhanced firm performance and sustained competitive advantage etc.) (Chuai et al., 2008, p.901; Iles et al., 2010, p.180; Dries, 2013, p.274; Cui et al., 2018, p.16).

As mentioned earlier, talent management sprout in a condition where classic human resources management system failed to meet the requirements of global business environment. Following the doctrines of resource based view (Barney 1991), and with the contribution of important researchers in the academic field (e.g. Chambers et al., 1998, p.45; Lepak and Snell, 1999, p.45; Axelrod et al., 2001, p.11; Axelrod et al., 2002, p.2; Collings and Mellahi, 2009, p.304; Bethke-Langenegger et al., 2011, p.527; McDonnell et al., 2011, p.177; Jones et al., 2012, p.413; Dries, 2013, p.273; Ewerlin, 2013, p.281; Valverde et al., 2013, p.1834; Sparrow and Makram, 2015, p.251; Gallardo-Gallardo and Thunnissen, 2016, p.44; Latukha, 2018, p.83–84; Meyers et al., 2020, p.580), we refer talent management as a more complex, sophisticated, systematic, and value-driven concept than classical human resources management, with its performance-oriented and differentiated structure.

The second dispute is related to the focal interest area of the talent management, whether it should be the whole workforce of the firm (inclusive approach), or just an elite group of high-potential, high-performing employees with superior contribution to business performance (Dries, 2013, p.279; Festing et al., 2013, p.1885; Bolander et al., 2017, p.1526; Tlaiss et al., 2017, p.428; Chung and D’Annunzio-Green, 2018, p.104; Crowley-Henry and Al Ariss, 2018, p.2066; Golubovskaya et al., 2019, p.4109). While exclusive TM concentrates on employees who take part in a selected, unique group identified as talents, inclusive TM considers talent as a thing that owned by all employees which could be discovered and developed with the help of necessary practices and processes (Gelens et al., 2013, p.348; Thunnissen and Buttiens, 2017, p.393; Kravariti and Johnston, 2020, p.82; De Vos and Dries, 2013, p.1817; Thunnissen, 2016, p.60; McDonnell et al., 2017, p.97; Crowley-Henry and Al Ariss, 2018, p.2065).

Al Ariss et al. (2014, p.176), claims that TM processes and programs could be better implemented on a target group as they able to receive the objectives as intended, accordingly, exclusive TM strategies are more competent in generating a congruous pool of employees. On the other hand, Iles et al. (2010, p.182) asserts that, in an ideal company every employee has a role to play and something to contribute, so inclusive TM approach is necessary in the revelation of the unique talents in all employees and improving the performance of whole workforce. In light of these discussions, a third option seems more reasonable. Stahl et al. (2012, p.26), revealed that the two approaches of TM are not mutually a single thing, many of the companies use a combination of both. The hybrid approach of the TM can be identified as a holistic perspective to talent management, in which the two distinct processes proceed simultaneously: a) labelling the entire workforce as organizational talent and assigning them as the focus of the developmental activities in line with inclusive approach; b) identifying the key and pivotal positions which pre-eminently make contribution to the company’s sustained competitive advantage and filling them with high-potential, high-performance, competent employees congruent with exclusive approach (Thunnissen et al., 2013a, p.1750; King and Vaiman, 2019, p.196; Marinakou and Giousmpasoglou, 2019, p.3866; Meyers et al., 2020, p.581). Stahl et al. (2012, p.26), also mentions that, by implementing a hybrid approach companies can differentiate and distinguished from their competitors and skirt the controversial issue of whether some employee groups are more valuable than others or not.

### Talent management in the context of Turkey

According to Tatoglu et al. (2016) context specific TM research has focused on a limited number of different countries (e.g. China, India, Oman, Vietnam, Spain, Poland, France, Germany etc.) and despite these numerous studies, there is a need to examine further the contextual nature of TM. And they highlight the lacuna existing in the general understanding of TM by emerging markets perspective. Turkey is the 19th biggest economy in the world and it is a labour-intensive market. According to the ownership of world fleet Turkey is ranked 16th by carrying capacity in deadweight tons. And it is also ranked 20th in Top 25 ship-owning economies (UNCTAD 2020). As an emerging market, Turkey hosts so many foreign direct investments and especially global shipping companies make extensive ventures for their branches in this country. In addition, as a nation with an organizational culture that synthesizes both western and eastern belongings, it attracts attention among other emerging countries. Organizational culture plays a fundamental role in shaping the TM structure, it limits and shapes the scope of the representative organizations’ TM system (Tatoglu et al., 2016). Demirbag et al. (2014) state that, Turkey is not just and emerging market, but also it is a country that where TM is becoming of prime importance in the national business environment which is in the seek for skilled labour and leadership talent. Confirming this information, The Talent Shortage Survey of 2018, conducted by ManpowerGroup, an U.S.-based human resources consulting company, involving six different industries and 39,195 employers, has pointed Turkey as the second most talent deficient country in the world after Japan with a talent shortage percentage of 66%.

Demirbag et al. (2016) assert that, multinational companies (MNCs) play a substantial role in the evolution of talent management concept in Turkey. Local companies do form international partnerships in order to extent their knowledge related to TM oriented tools and techniques. Often these western-style HRM practices are transferred to the country through multinational companies, even if they compete with local firms for the same talent (Wasti, 1998, p.625; Demirbag et al., 2016; Tatoglu et al., 2016). Tatoglu et al. (2016) claim that, there is evidence that Western HRM practices have been embraced amongst local firms in Turkey but this development remains in a nascent state. However, some opposing views claim that local Turkish firms are mostly family-run businesses, and therefore a tribal approach and a system of placing family members at key points is adopted instead of modern approaches such as talent management (Kaya, 2006). Moreover, it is claimed that even if these companies try to implement modern HR systems with the pressure of the developing and changing business environment and the influence of their external stakeholders, these initiatives are not gone beyond trying to imitate Western-style systems without considering their own organizational dynamics (organizational culture, employee portfolio etc.) (Tuzuner, 2014, p.448; Tatoglu et al., 2016). Correlatively, Tatoglu et al. (2016) have alleged that, it is necessary to examine whether there is a culturally specific and relevant meaning for talent and talent management concepts among Turkish HR executives, and then further assess whether these meanings are consistent with practice. In addition, they have claimed that the examination of talent and talent management concepts together with general approaches and debates, within a combined sample of local Turkish companies and multinational companies, could show if there is a difference between their views and interpretations regarding these issues and they have argued that this could be an answer to the previously mentioned discussions. Taking these arguments into account, the sample of the study has been organized as a combination of overseas-based multinational, Turkish multinational, Turkish large-scale, Turkish medium-sized and joint venture container shipping companies operating in Turkey (see Table 4 in Appendix 3).

### Talent management in shipping

Meyers et al. (2020) claim that, empirically investigating the existence and handling of talent management related approaches and debates from different country and industry contexts is a necessary step in understanding the concept as a practical phenomenon more thoroughly. Pantouvakis and Karakasnaki (2018), have taken notice this inducement and they have investigated talent related approaches in the shipping industry for the first time. In this pioneer study they have investigated innate vs acquired and inclusive vs. exclusive approaches within the shipping sector. However, there are other streams in the talent management literature that are considered among the main comparative trends of talent oriented approaches (object vs. subject, input vs. output, transferable vs. context-dependent). Furthermore, a major debate centring the relationship between human resources management and talent management is still questioning by the researchers. Accordingly, following the referral of Meyers et al. (2020), it is thought that researching these issues in the container shipping industry from an emerging market perspective could bring a newer view and create interesting insights.

Despite the shift towards the capital-intensive paradigm, the human capital is still recognized as one of the most valuable and unique assets in the shipping industry. When it comes to the container shipping industry, the key role of valuable and rare human capital is undeniable (Parola and Satta, 2012; Haralambides, 2019; Notteboom et al., 2019). The dynamic and competitive nature of the container shipping leads companies to recognize the importance of having qualified, valuable, competent and unique human capital to enable them sustaining excellence in the provision of customer-focused services and accomplishing business objectives within the industry (Ng et al., 2009, p.257; Pantouvakis and Karakasnaki, 2019, p.277). Progoulaki and Theotokas (2010) consider the differentiated and distinctive management of human capital as the primary requirement for sustainable competitive advantage, taking into account the firm’s resource-based view. Chambers et al. (1998: 44) defines talent as the unique and valuable human capital of a firm and also talent management is defined as a concept directly related to the specific and exclusive management of talent (Iles et al., 2010, p.135; Bethke-Langenegger et al., 2011, p.527). In this context, talent management (TM) can be considered as an important factor contributing to the achievement of corporate goals in the shipping industry and effectively influencing the successful implementation of business strategies through the way of sustained competitive advantage (Pantouvakis and Karakasnaki, 2018, p.649). According to the Pantouvakis and Karakasnaki (2018), defining talent and TM and further investigating related approaches and contradictions within shipping industry is vital because it is a sector that requires high human competencies and skills.

Even a simple web search will show that talent management has been identified as one of the strategic priorities in industry-leading shipping companies and their talent management related knowledge and expertise is recognized and remunerated in the global scale. One of the research aims of our study is to examine the details of this priority by analysing and synthesising the regarding opinions of human resources officials acting in the sector leading companies, in order to establish a working-definition of talent and talent management concepts which will create an insight for and will guide companies that will enter the sector or try to strengthen their existing positions. For example, Maersk Line which is also a participant within the sample of our research was named by The Association of Talent Development (ATD), as “world’s best organization for talent development” and won the 2015 and 2016 BEST Awards consecutively thanks to the success of their talent management system which is very high on the Maersk agenda. In addition, another participant within the sample of our study, DHL, which is also a company functioning within the container shipping industry that highly notices talent management, has been certified as “Top Employer Europe” and “Top Employer Turkey” in 2020 by The Top Employers Institute which is a certification programme that enables organisations to assess and improve the workplace environment. These developments reveal that the actors in the container shipping industry directing the field of practice in the context of talent management and have the potential to substantially contribute to the development of the concept.

## Methodology

Phenomenology method has been used as research design of the study. Phenomenology research design aims to reveal the original ideas and interpretations of an individual about the phenomena (social conditions/events, business processes, etc.) that he/she experiences and observes throughout his/her working life from the perspective of the researcher (Bloor and Wood, 2006, p.128). Interview data collection method is a suitable tool to be used in phenomenological studies to understand and interpret the ongoing phenomena that are shaped around the interviewee (Sığrı, 2018, p.186). In this context, on the basis of research questions and research design, open-ended questions obtained from the relevant literature has been organized in a structured interview format and applied to the human resources officials working in the logistics industry, after necessary translation and content checks has been accomplished.

Interview is a data collection method that aims to understand individuals and facts related with them, through verbal communication. The main purpose is to collect qualified data on the research subject (Sığrı, 2018, p.237). In structured interviews, researchers conduct the interviews in a standardized manner by asking all the participants pre-organized and open-ended questions in the same order. The strength of such interviews is that they provide consistent data production and the ability to make appropriate comparisons between participant’s responses (Van Niekerk and Savin-Baden, 2010, p.33; Savin-Baden and Howell-Major, 2013). In the interview method, validity is determined by expert opinions. This method proceeds over steps like: the framework of the research, data collection form, interview conduct guide and asking questions to the participants. The comprehensiveness of the data collection tool specifies the validity and the similarity of the results in process of the repetition states the reliability, which is evaluated through credibility, transferability, dependability, confirmability and integrity (Wallendorf and Belk, 1989, p.71–72). After all these reliability and validity considerations has been accomplished and the related data has been collected, the obtained data have been analysed by a qualitative content analysis method.

Content analysis is the systematic and objective examination of the obtained data’s content. The main purpose in content analysis is to reach the concepts and relationships that will enable to explain and interpret the collected data (Roberts, 1989, p.148; Krippendorff, 2004, p.88; Roberts, 2015, p.769). Transcribed written interview texts are usually the starting point in qualitative content analysis. Analysis of the raw data from literal quoted interviews to organize categories or themes is a process of further condensation of data at each step of the analysis; from the explicit content to veiled meanings. Qualitative content analysis basically proceeds on five consecutive phases: identifying meaning units; condensing meaning units; coding; forming categories and theme building (Creswell, 2007, p.148; Mayring, 2014; Erlingsson and Brysiewicz, 2017, p.93–94).

MAXQDA 2020 qualitative content analysis software has been used to analyse the data obtained via interviews. MAXQDA provides a more systematic way of data analysis compared to manual works (Kuckartz and Rädiker, 2019). During the analysis process, two coders have been selected among the experts who participated in similar studies and they have received training to encode in order to increase reliability between coders. Through the coding analogy of the encoders, it has been revealed that the Cohen’s Kappa coefficient scores are not less than 0.75. This situation demonstrates that there is an agreement between the encoded texts (McHugh, 2012, p.279). If both encoders have completed agreement on which content to encode, the Kappa coefficient is 1. If there is no consensus between the two implementers (except for coincidental events), the Kappa coefficient is 0. The value between 0 and 1 demonstrates partial agreement. In this case, it can be reached to the implication that content analysis of the study has reached agreement (Kuckartz and Rädiker, 2019, p.281). An inductive approach has been embraced in the coding and analysis processes. The use of an inductive approach allows the participants’ tendencies to be identified and transferred to research results rather than the researcher’s ideas.

The coding paradigm of Corbin and Strauss (1990) has been taken as the methodological basis. The coding of the data has been begun with open coding and then the axis codes have been combined. Later on, the research has been themed by selective coding. Along the coding process, the data has been read repeatedly, and the primary codes has been generated. Interview questions have been initially handled as an umbrella category, then as codes emerged, interrelated codes have been categorized under themes. Following, the themes obtained have been attempted to be expressed via visual analysis in a manner that the readers could understand. Finally, the findings have been commentated in order to give meaning to the findings obtained through the analysis. An example of the code and category creation process is shown in Table 1.

**Table 1:** Coding and categorization example in data analysis process

Question: What does the concept of talent management means for your company?				
Theme	Category	Code	Raw Data	Researcher’s Interpretation
Talent Management	Definition of Talent Management	Development	‘*Talent management refers to: the work done for the development of employees identified as talent; the additional responsibilities given to these employees; the arrangement of the plans for bringing in greater visibility to talents within the company and enabling them to take on bigger roles in the future.*’ (P17)	Participant refers TM as the efforts proceeded for the professional development and potential display of talents.

In the process of reporting the findings, firstly, the main themes have been presented. Later, theme-based categories have been explained via the support of visuals and the findings have been interpreted. In organization of the findings, the relationships between the codes have been demonstrated, a holistic meaning has been developed by establishing cause-effect relationships and making conclusions from the obtained findings.

The data have been collected via online, structured interviews, and the data collection tool has been developed via the questions gathered from the relevant literature. The form has been critically analysed by five academicians from a variety of institutions (1 professor from University of Piraeus, 1 assistant professor from Warsaw University of Technology, 1 professor and 1 associated professor from Dokuz Eylul University and 1 assistant professor from Istanbul University), in regards to whether the questions are comprehensive and proper to obtain the aimed data to accomplish the reliability concern. Besides, to test the validity of the construct, five human resources specialists have been interviewed utilizing the organized form, and the interviews were repeated 1 week later to control whether the responses would be the identical or not. Receiving similar answers ensured that the data collection tool has the expected validity.

Purposeful (purposive) sampling is a method that is typically used in qualitative research for the identification and selection of knowledge-intensive samples in order to evaluate limited resources effectively. This involves designation and selection of individuals that are especially acquaintance with or experienced in a relevant phenomenon. In addition to knowledge and experience, availability and willingness to participate are also important criteria within the purposive sampling (Palinkas et al., 2015, p.534). Through this extent, 27 HR executives from 21 different logistics companies (see Table 4 in Appendix 3), have been designated as the research sample via purposeful sampling. Data were collected between May and November 2020. The pandemic conditions caused by COVID-19 have not allowed the research conducting face-to-face, all interviews have been conducted via online. The interviews have lasted for an average of 35 min and it has not been possible to record audio from most of the interviews due to the privacy concerns of the companies. For this reason, the opinions of the participants have been carefully and verbatim transcribed to the written text by the interviewer. The participants of the research are numbered from P1 to P27 and the profile info of the participants are given, in Table 4 in Appendix 3.

Finally, as stated earlier, robustness and comprehensiveness in qualitative studies are generally provided by expert opinions (Wallendorf and Belk, 1989, p.71–72). In this context, a form containing questions about the industry-specific validity of the findings has been developed in order to test the robustness and comprehensiveness of the main findings together with policy implications. This form has been sent to 10 of the interviewees, which have been chosen according to their expertise in the field and activeness in the industry, to collect their comments and to gather deeper, better insights regarding the research findings. These experts have been determined by purposive sampling through the reasons explained in the previous sections. It is asserted that, these types of interviews are useful tools for fully understanding a new phenomenon, such as developing a new context-dependent definition. Such researches are also claimed as helpful for the robustness check of the findings and policy implications in qualitative studies (Sekeran and Bougie, 1976).

## Findings

### How do the representatives of container shipping companies operating in Turkey define talent phenomenon?

Participants have heavily stated the meaning of talent as *competency/capability*. According to the participants, *competency/capability* reflects the high performance and potential of the individual and to be sufficient in the works he/she has to do:

“Talent is the competency a person demonstrates in performing something.” (P5)

“Talent is an individual's specific combination of predisposition and competencies.” (P27)

Another frequently repeated expression that is put in the centre while defining talent is *developable*. Participants refers talent as something can be developed in all employees together with the right training and education programs:

“Talent is the potential of individuals which is developable and can be transformed into competence with the necessary training and guidance.” (P8)

“Talent is the potential of an employee to use and develop his/her knowledge, skills and abilities correctly.” (P24)

Another term has been expressed for the definition of talent is *innate*. Talent has been described by the participants as the innate capacity and potential of the individual:

“Talent is the innate power and capacity to adapt to a situation and to be successful.” (P16)

“Talent is the innate ability of individuals to learn, do business, understand and perform an action.” (P27)

As in the definition of talent, competency is again the most commonly used concept when describing who is considered as a talent. Employees who can positively contribute to the business goals of the company with their knowledge, skills, abilities (KSAs) and competencies are considered as talent:

“Talent, refers to the employee who is disciplined in his/her work and have the capabilities required by the job, and who have the necessary knowledge, skills and abilities not only in his/her speciality but also in other work related fields.” (P25)

“Employees who can make a difference with their potential and competencies can be considered as talent.” (P6)

Another concept has been emphasized by the participants when explaining who is considered as talent is to create value for the company and to contribute to the company benefits. Employees who contribute positively to the interests of the company and create value in order to improve business performance are considered to be talent:

“Employees who conduct research and examinations and use them for the benefit of the company, who have a passion for success, develop new ideas, are always open to learning and development are talent.” (P7)

“Anyone who can exceed the expected efficiency, contribution and added value with the assumption that performance goals are correctly determined and evaluated can be referred as a talent. Talents are determined by evaluating different parameters such as continuous personal development, vision, and willingness to learn.” (P21)

Other term which has been frequently denoted by the participants in order to express who is accepted to be talent is uniqueness. According to the participants, uniqueness is the ability of the individual to fulfil the assigned tasks differently from everyone else, in other words, the ability to make a difference and being rare. So that, employees who can perform tasks different than others and in a unique way are accepted as talent:

“People who do their job uniquely and create a good comparative difference are talent.” (P2)

“Those who have a certain level of education and experience, can do their jobs in a different way, in a shorter time and more easily are talent.” (P18)

### What does the concept of talent management mean for the container shipping companies functioning in Turkey?

According to the participant statements it is seen that *development* is the primary term utilized to reflect the interpretation of talent management notion in the Turkish container shipping industry. Participants have expressed the meaning of talent management as identifying, training and developing talents according to business objectives and they also emphasized the importance of investing in the development of internal talents:

“In our organization TM is defined as the effective implementation of all necessary specialized development processes in order for our employees to reach their maximum potential and performance targets by accepting all of our manpower as talent.” (P10)

“Talent management can be summarized as discovering talents from the company's internal resources and investing in their development.” (P27)

*Competency-oriented deployment* is an another concept which is frequently emphasized to define the notion of talent management by the participants. Participants have stated that assigning the right individuals to the right tasks has a significant effect on performance and it is the primary ingredient of talent management concept:

“Talent management can be expressed with the phrase of “the right person for the right job”. Even a person may be very talented, they may not perform adequate at the job that does not fit their character and attitude. For this reason, the quality of the job should be analysed well and individuals should be selected and deployed according to the job.” (P11)

“Talent management strategically means having the vision of placing the right people, at the right time and in the right positions in the organization, and creating a specific talent pool and succession plan for situations that may arise in the future.” (P17)

The concept of *retention management* appears as another term substantially expressed by participants when defining the notion of talent management. Participants have asserted that efforts to increase the loyalty of individuals (whose talent has already been discovered and developed) to the company by supporting them with in-house promotion, valuation and rewarding programs are the basis of talent management:

“Talent management refers the work done to prepare an environment where talented employees can work more efficiently and the efforts to retain them.” (P18)

“Talent management is a set of systems that covers the entire process of identifying talented employees, acquiring them to the company, supporting their development according to their competencies, deploying them in appropriate positions and more importantly retaining them with the support of well-established plans.” (P23)

### From what dimensions are talent and talent management concepts approached in the industry?

When the status of the basic approaches to talent and talent management is examined in the Turkish container shipping industry, it is seen that an inference can be reached for every stream. When the views of the participants on talent are examined in terms of the subject vs. object approach, it is seen that although talent is expressed as a phenomenon by some of the participants, it is generally considered and expressed as an individual in the Turkish container shipping industry. This situation indicates that the dominant approach in the industry regarding this debate is the subject approach. As can be seen in the views of the participants on the definition of talent, talent is mostly defined as an inborn power or ability. This finding reveals that the prevailing view in the shipping industry is towards the innate approach. However, unlike the tenets of current literature, in Turkish container shipping industry talent is evaluated as an inborn but also developable belonging. This development is not explained as creating from scratch, but as carrying the existing phenomenon to an advanced level with the necessary effort. The fact that the dominant concepts in the definition of talent are competence and capability notions, and the central point of the answers given to the question about who is considered as talent by the participants is to contribute to the interests of the company, shows that the output approach is popular in the container shipping industry in terms of discussions regarding the input vs. output approach. When the participants’ views on talent has been examined, it has been seen that adaptation concept has repeated more than once. It is stated that the talent can easily adapt to the conditions around him/her and can easily demonstrate his/her potential, competencies, capabilities in every field, regardless of the environment he/she is in. In this context, in terms of the transferable vs. context dependent approach, it is understood that the basic view in the Turkish shipping sector is in favour of the transferable perspective.

Participants have advocate that each of the employee who have been brought into the company as a result of well-designed processes has a unique talent and that their potential can be revealed with the right guidance and developed with the necessary training and education programs. The participants have also stated that talent management includes all kinds of activities performed to reveal the potential of the existing employees and this situation provides the benefit of the company. As a result of all this information, it would not be wrong to say that all workforce is considered as potential talent in Turkish container shipping companies and that talent management is usually approached with an inclusive perspective. However, the existence of both the exclusive and inclusive approach are felt in a balanced way in overseas-based multinational companies.

### How is the relationship between talent management and human resources management interpreted by the container shipping companies?

By far, the most frequently repeated concept by the participants is TM as a sub-process of HRM regarding the HRM vs. TM debate. Most of the participants have emphasized that talent management cannot be considered as a separate process from human resources management, on the contrary, it is a sub-process of HRM:

“Human resources management is concerned with not only human talent, but also all human characteristics including talent management according to all specifics of the business.” (P20)

“Talent management is a sub-process of human resources management. Human resources management comprises and handles all labour related issues within the company.” (P24)

One of the other concepts emphasized by a group of participants in order to express the difference between talent management and human resources management is career development and planning. Participants have defined talent management as a system that focuses on talented employees, unlike human resources management, and reflects the entirety of the practices carried out to identify talented employees and plan and develop their careers within the company in a way that contributing to the accomplishment of business objectives:

“Unlike human resources management, talent management focuses on what can be done for the acquisition, development and retention of talents and specifically deals with the career development and planning of talented individuals.” (P14)

“Different than the human resources management, talent management deals with the management and organization of a limited number of unique personnel that can directly create added value in the form of intellectual capital through specific practices such as competency-oriented career planning.” (P27)

The last context where the differences in talent and human resources management are expressed by the participants is professional development. The participants have stated that unlike human resources management, each employee is given special attention in talent management and that the professional development of the employee is focused. Participants have identified human resources management as a system focused entirely on procedures, instead of employees, while talent management has been interpreted as a human capital oriented notion that is based on the most accurate development of talent, which is an original, valuable and inimitable asset facilitates the achievement of business goals by contributing to the company’s competitive advantage:

“Talent is extraordinary and unique, so they should be included in a professional development plan separate from standard human resources practices. These talent-oriented plans and systems are gathered under the concept of talent management.” (P8)

“In talent management, the requirements of the job are matched with the competencies of the employee and the differences between them are tried to be developed. Unlike human resources management, employees are not only considered as a tool, but as potential talents that are of great importance in achieving the company's goals. Tailor-made professional development practices carried out in order to bring this unique human power, which is of great importance to the company, to the highest level of its potential constitute the heart of talent management.” (P13)

### Further inferences

**Fig. 1:**
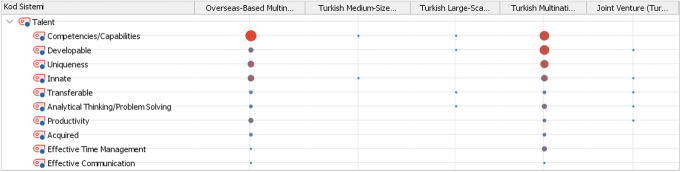
Expressions of talent via company types

**Fig. 2:**
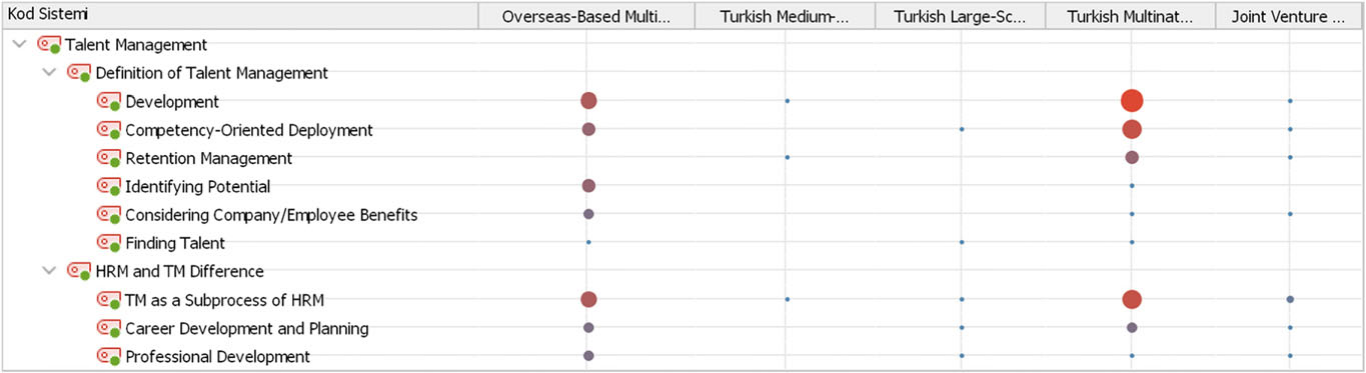
Expressions of talent management and HRM-TM difference via company types

The research shows that human resources officials working in multinational companies provide more intense, detailed and fundamental views on talent and talent management notions and have a better understanding of the concept (see Figs. 1 and 2). When the talent theme is examined according to company types, it can be seen in Fig. 1 that overseas-based multinational companies primarily focus on *competencies/capabilities* notions. This inference reveals that overseas-based multinational companies operating in the Turkish container shipping industry generally approach the concept of talent from an output approach and attach importance to concepts such as competency, capability and productivity that directly related to the performance of their employees. Employees are expected to be competent not only in their own expertise, but in all areas, and to contribute to the interests of the company and create value.

Although, the concept of competencies/capabilities is also frequently included in the expressions of Turkish multinational companies, it is seen that the term developable is the most intensely emphasized notion by the participants working in these companies. It is seen that the concept of development is not expressed intensely in overseas-based multinational companies. This situation stands out as the most obvious difference between Turkish and overseas-based companies in terms of talent concept. Another striking difference in the approaches of multinational companies and other Turkish companies towards the concept of talent occurs in the term of *uniqueness*. It is an interesting observation that while the concept of “uniqueness” is often attributed when defining talent in multinational companies, it is not mentioned at all in Turkish companies other than multinational companies. Uniqueness has been explained as the ability of the individual to fulfil the assigned tasks differently from everyone else, in other words the capability of making a difference.

When a general approach is desired to be reached in the light of the relevant findings, it is understood that talent is defined in the Turkish container shipping industry as innate and developable features (e.g. competencies, capabilities etc.). Besides, individuals who are competent in their field and can complete their duties in a unique way by making a difference are accepted as talent.

When we examine the theme of talent management according to company types, it is seen that talent management in the Turkish container shipping industry is evaluated in a broad perspective. As can be seen in Fig. 2, in almost all types of companies, talent management is primarily interpreted with the concept of *development* and they evaluate talent management with an *inclusive approach*. Competency-oriented deployment is another highly emphasized concept utilized in the industry for the definition of talent management notion. It is observed that the main difference between Turkish and overseas-based companies in their approach to the talent management concept is manifested through retention management. Although, it is often attributed to retention management while defining talent management in almost all types of Turkish companies, it is noteworthy that this concept is not mentioned at all by overseas-based companies. Participants working in Turkish companies stated that the efforts to increase the loyalty of individuals whose talent has been discovered and developed, by supporting them with versatile and comprehensive talent retention practices (e.g. in-house promotion opportunities, work-life balance arrangements, merit-based remuneration packages etc.), constitute the basis of talent management. On the other hand, another absorbing difference is that although the concept of *identifying potential* is frequently mentioned when describing talent management in overseas-based companies, this concept is almost never used in Turkish ones. Participants operating in overseas-based companies have stated that talent management includes all kinds of activities performed to reveal the potential of the employees and this situation provides the benefit of the employee and/or the company.

The interpretation regarding the difference between talent management and human resources management in the industry is intensely expressed by the *TM as sub-process of HRM* concept. Especially Turkish companies evaluate talent management as a concept within human resources management and defines it as a sub-process that deals with a specific part of the workforce. However, talent management is defined as a completely different concept from human resources management with its focus and approach to employee, especially in the case of overseas-based multinational companies.

## Discussion

It is often emphasized by researchers that there is no universally accepted definition of either talent or talent management in the literature yet (Ewerlin and Süß, 2016: Harsch and Festing, 2020). The main reason for this fact is claimed to be that these concepts are context-dependent and that they are evaluated and defined according to the own variables and dynamics of each environment (Al Ariss et al., 2014; Krishnan and Scullion, 2017). In addition, it is asserted that the current definitions of talent and talent management are conceptually created without being based on an empirical research and are far from reflecting the realities and views of the practitioners (Khoreva and Kostanek, 2019). In this regard, it has been frequently emphasized that it is an important requirement to consider, evaluate and define talent and talent management concepts from different contexts and different country perspectives for the development of talent management field (Pandita and Ray, 2018; Marinakou and Giousmpasoglou, 2019).

Considering all these alleged requirements, it has been thought that evaluating, handling and defining talent and talent management concepts within the Turkish container shipping industry could contribute to the development of the talent management field. One of the main reasons for this thinking is that talent management has been recognized as a strategic priority for industry-leading container shipping companies which are also included in the sample of the study, and their knowledge and expertise in talent management are recognized and rewarded on a global scale. Therefore, it is clear that the opinions of companies that directly influence and direct the development of the talent management centred practices field will also contribute to the theoretical development of the notion. Correlatively, Turkey, which serves as a bridge between eastern and western cultures, is being claimed as one of the countries with the highest need for talented workforce in the world, therefore, talent management has become the top priority in the country’s quest for skilled labour and leadership talent. In this direction, considering that these concepts are already examined in depth and in detail in the Turkish business world, which has made significant investments in talent management recently, it is thought that the opinions of its representatives will contribute to the development and conceptualization of talent and talent management notions.

In this context, after synthesizing the main themes developed regarding talent notion, it can be said that there appear to be two different common definitions of talent suitable for Turkish shipping industry. From the object view; talent refers to *the unique and developable competencies and capabilities (*e.g. *analytical thinking, problem solving, effective time management and communication, productivity, creativity* etc.*) which an employee already has and can be revealed with sufficient learning and education efforts*. From the subject approach; *employees who are highly motivated, open for development, competent in their field and can complete their duties in a unique, creative and productive way by making a difference and who can shine regardless of the working environment*.

Similarly, the definition of talent management, which can be embraced as a unanimous context for the Turkish container shipping industry, has been formed as a result of the themes obtained by combining the common codes created from the responses of the participants regarding the expression of talent management. Through this extent it has been observed that TM concept is defined in the container shipping industry as; *identifying high potential, competent candidates and employees together with key positions within the organization, systematically deploying talents to the critical positions after the required professional development processes have been accomplished and increasing the loyalty of high performing employees with in-house promotion, valuation and rewarding programs by considering both employee and company benefits*.

As it stated earlier, an additional process has been handled for the robustness check of the primary findings and policy implications. As a result of this additional process, it has seen that the main findings of the research are reflecting the realities of Turkish container shipping industry and the definitions developed are largely corresponding to the descriptions desired and predicted in the industry. Policy implications have been seen as extremely beneficial, and most respondents agreed with these suggestions. Participants have emphasized that the inferences made address extremely important problems and that solutions can help companies experiencing subject problems.

Investigating the existence and handling styles of talent and talent management related approaches and debates from different country and industry contexts have been claimed as a necessary step in understanding the concept as a functional phenomenon more comprehensively by Meyers et al. (2020). As an answer to this consideration, the study conducted by Pantouvakis and Karakasnaki (2018), emerges as a pioneering study examining the approaches and debates on talent and talent management concepts in the shipping industry. However, they have examined only one of the main trends towards talent-based approaches in their research (innate vs. acquired). Additionally, of the two main TM-centred discussions, they have only mentioned and investigated the debate related to exclusive vs. inclusive approach. In this sense, one of the aims of the study has been determined as taking their vanguard work a click forward and examining all of the talent and TM-oriented debates in the context of Turkish container shipping industry.

The in-depth analysis regarding how talent is interpreted in the container shipping industry demonstrates that the concept of talent is handled with both an object and a subject approach. As it is mentioned in the literature, subject approach considers talent as high potential and competent employees and object approach evaluates talent as characteristics of employees such as capabilities and competencies (Thunnissen et al., 2013b, p.327; Mensah, 2015, p.548; Bolander et al., 2017, p.1525). However, research findings show that the subject approach is gaining more acceptance in the container shipping industry and the meaning of talent is heavily expressed by considering an employee. It is claimed by Dries (2013), that putting the subject approach to the fore too much, will cause talent development practices such as, competence and knowledge management to be ignored. Vance and Vaiman (2008, p.7) allege that knowledge management increases the perceived importance of talent management strategies, practices for the organization and provides a common unifying purpose to more effectively integrate and coordinate these components with various talent management functions. In order to solve this problem, it may be suggested that companies operating in the Turkish container shipping industry give more value to the object approach of talent. In this way, they can improve their intellectual capital by giving more importance to system-oriented practices such as knowledge management. Shaikh defines intellectual capital as the knowledge that can be transferred into value. It has been asserted that intellectual capital has various benefits for companies, such as, facilitating innovation, enhancing employee competencies, gaining competitive advantage, improving organizational performance and increasing company reputation (Gogan et al., 2016; Ginesti et al., 2018; Yıldız et al., 2018). During the robustness check, only one participant disagreed with this view and stated that employees form the basis of talent management, therefore a system approach independent of individuals cannot be considered. However, it has been argued by the rest of the participants that the opinion regarding the dominance of subject approach in the container shipping companies is correct and that systems approach that consider talent from a factual view is a necessity.

The description of talent by the participants mostly centred on the notions of competence and capability, correlatively, this inference can be evaluated as an evidence for the dominance of output approach in the shipping industry. Because, according to Dries (2013) output approach focuses on employees’ competencies and capabilities and it qualifies talent as an employee’s performance, achievement and contribution to the company. Likewise, the HR executives employed in the container shipping companies have asserted that, *competency/capability* notion reflects the high performance and potential of the individual and to be competent in the tasks he/she has to do. According to Ulrich and Smallwood (2012), talent is a combination of commitment and competence. They argue that the balance between these two components should be preserved, because any reduction in the two components will affect also the other one negatively. In short, they claim that an employee with a decrease in motivation and determination will not be able to provide the desired performance and contribution. This situation reveals the fact that, if the Turkish container shipping companies keep on favouring output approach by disregarding the motivation and ambition of the employees, they would not be able to get the desired performance and contribution from their talents. This inference has gained a full acceptance along the robustness check.

According to Dries (2013), the innate approach argues that talent is inborn and cannot be taught, whereas acquired approach asserts that, talent can be learned and developed. Our study reveals that, in the container shipping industry talent is considered as an innate but further developable belonging. This view is something like the hybrid form of innate and acquired approaches. This finding coincides with the approach that Nijs et al. (2014) have mentioned when defining talent. Nijs et al. (2014) describe talent as innate abilities of individuals that can be systematically developed and deployed in the right activities of interest. Similarly, by the participants of our research, talent is accepted as an inborn power but also it is thought that it could be enhanced and polished. Cappelli (2008) states that, the pure innate approach can be challenging for the organization in a talent deficient environment. This situation presents that Turkish container shipping companies are aware of their business environment’s deficiencies and take precautions by developing hybrid approaches. The hybrid approach which broadly utilized by Turkish container shipping companies can be proposed as a solution for areas with high skill needs. This argument has been supported by the participants during the robustness process, and in particular, it has been emphasized that many companies provide opportunities for young talents who have recently graduated from the maritime and logistics departments of universities. Additionally, they have stated that, after attending in necessary training programs and gaining experience, these talents are generally evaluated in critical positions.

Container shipping companies operating in Turkey, evaluates talent as something transferable. Transferable approach postulates that talented people can demonstrate their potential and performance regardless of the working environment (Dries, 2013, p.280). Dries (2013) argues that thinking that talent can exhibit full potential and performance under all conditions will cause some mistakes. This way of thinking can prevent companies from making the necessary contextual improvements to increase the performance of the talent. Considering that talent will contribute directly without any effort, companies may ignore practices such as orientation procedures, employee friendly work environment arrangements, customized training and education programs. The robustness check has supported the accuracy of this inference, and most of the participants have expressed that they think this problem as an issue that needs to be worked on. Accordingly, it is recommended companies, to handle talent by context-dependent approach more, to take necessary steps and create required systems together with conditions in enabling employees to shine comfortably and show better performance.

From the participant views, it has been observed that in the container shipping industry, talent management is usually handled with an *inclusive* approach especially by Turkish multinational companies, inclusive TM considers talent as a thing that owned by all employees which could be discovered and developed with the help of necessary practices and processes (McDonnell et al., 2017, p.97; Crowley-Henry and Al Ariss, 2018, p.2065). While an inclusive approach to talent management is believed to lead to a more pleasant working environment characterized by openness, trust, and overall employee wellbeing which serve as a catalyser of motivation and determination, the exclusive approach is assumed to generate higher return on investment in terms of profit and productivity (Boudreau and Ramstad, 2005; Warren, 2006; Dries, 2013). To get these gains together by using a combination of the two approaches seems more reasonable for container shipping companies. Stahl et al. (2012, p.26), claims that, by implementing a hybrid approach companies can differentiate and distinguished from their competitors and skirt the controversial issue of whether some employee groups are more valuable than others or not. As stated in the previous sections, indications of both exclusive and inclusive approach are felt in a balanced way in the overseas-based multinational companies within the sample. Confirming this indication, Maersk which is a globally remunerated, leading company of the industry and a participant of our study, states that they implement a balanced hybrid approach with a motto of “build, buy or borrow”. They comment that this hybrid approach prevents them to rely solely on internal talent, and it helps them to enhance their talent portfolio with fresh eyes and energy (Groysberg and Abbott, 2012). During the robustness check, the presence of such a problem has been accepted by the participants and the idea of applying a hybrid approach (combining inclusive together with exclusive view) as suggested has been considered as needful and helpful. It is thought that, for Turkish container shipping companies, adopting a hybrid approach (inclusive with exclusive) to talent management, like their overseas-based competitors in the industry, will increase their competitive power. While organizing specialized programs with an exclusive approach for the critical positions of the company, general talent management programs can be implemented for the whole workforce of the company with an inclusive approach. This helps them differentiate from their competitors and bypass the controversial issue of whether certain groups of employees are worth more than others. At the same time, the continuous development of the company’s existing talents is ensured, while enriching the talent pool with fresh energy and insights that may be required.

The distinction between talent management and human resources management is substantially stated by the *TM as sub-process of HRM* concept in the container shipping industry. Especially Turkish companies evaluate talent management as a concept within human resources management and defines it as a sub-process that deals with a specific part of the workforce. In contrast, it is seen that the concept of talent management is identified as a completely different concept from human resources management with its focus and approach to employee, especially in the case of overseas-based multinational companies. This view argues that, while human resources management handles employees as a tool and focuses in procedures; talent management attaches importance to systematic functions such as identification, acquisition, development, deployment and retention of talent, which is a unique, valuable and inimitable asset that will directly contribute to the company’s business objectives and competitive advantage. These observations draw a framework that supports the argument made by Tatoglu et al. (2016), that Turkish companies follow modern approaches from behind and rely on their foreign partners to develop modern applications.

Kabasakal and Dastmalchian (2001) claims that, Turkey is considered to be one of the most collectivist societies in the world. Additionally, Valverde et al. (2013) argue that companies within collectivist cultures form TM programs with an individualistic and exclusive approach. However, the findings of our research postulates that, especially Turkish multinational companies in the industry by embracing an inclusive approach see talent management as something that can be applied to the entire workforce and that development practices and processes are at the centre of this system. “*Right person to the right job*” motto has been embraced and identification of key positions and the systematic deployment of talents to this positions through competency and potential has been accepted as a central context within the talent management system which mostly reflects the TM construct. This finding support the view put forward by Demirbag et al. (2016) that multinational companies play a substantial role in the evolution of talent management concept in Turkey and international partnerships direct the TM-oriented approaches of Turkish local companies.

## Conclusion

Even, empirical researches have begun to increase in the talent management domain, various researchers mention the inadequacy of experimental studies and, talent management (TM) is still claimed as a young and developing field (Cappelli and Keller, 2014, p.306; Cooke et al., 2014, p.226 Festing and Schäfer, 2014, p.268; Tlaiss et al., 2017, p.442–443; Jooss et al., 2019, p.2). It has been asserted that TM research is dominated by Anglo-Saxon countries, and this fact bears the need for an intense contribution from the rest of the world to totally command on different perspectives of talent management concept (Valverde et al., 2013, p.1832; Gallardo-Gallardo et al., 2015, p.268; Gallardo-Gallardo and Thunnissen, 2016, p.36; Thunnissen, 2016, p.58; Marinakou and Giousmpasoglou, 2019, p.3861; Pandita and Ray, 2018, p.188). In addition, TM researchers state that, investigating the TM considerations in different industries and emerging markets is an essential necessity for the development of TM field (Farndale et al., 2010, p.1056; Latukha, 2015, p.1056; Latukha, 2018, p.73).

This study reveals its importance as a response to all these needs mentioned. The research is a unique work that investigates TM related concepts within the container shipping industry. Furthermore, these concepts are also seldom studied within the shipping industry. Also, it is one of the a few studies that investigates these considerations from a Turkish perspective which is considered as an emerging market. This study contributes to general theory with the different perspectives and insights of, an industry that is effective in the talent management centred field of practice, and a country that has made considerable investments in talent management recently. And it also provides a resource where companies in the Turkish container shipping industry can analyse their current positions and benchmark their own views with the attitudes across the industry.

For the theoretical development of talent management, the empirical examination regarding how the components of the concept are defined in the field of practice is often alleged as a necessity. From this point of view, it is thought that defining the relevant concepts in the container shipping industry, which has globally recognized and remunerated representatives in the field of talent management as well as in world trade, could bring some new perspectives and insights to the general theory of talent management. Relatedly, definitions that can be accepted as unanimous for the industry have been developed by compiling the opinions obtained from a sample including the leading representatives of the sector under common themes. The comprehensiveness and validity of these definitions in the context of the industry have been critically discussed with the selected interviewees. And as a result of this procedure, after organizing minor revisions, it has been found that the definitions developed substantially reflect the realities and general perspective of Turkish container shipping industry.

One of the biggest limitations of the study was the difficulty of meeting face to face due to the pandemic period. This situation also made it difficult to reach the relevant managers and greatly reduced their willingness to participate in the study due to conditions that place more workload on human resources executives such as part-time working arrangements, and special insurance practices, so the data collection process in the study has taken a relatively long time. Future studies could expand the scope of research across the entire shipping industry comparatively in different countries, including both the liner and tramp sectors. By this means, a general understanding synthesises maritime business and talent management fields can be obtained.

## Data Availability

The datasets used and/or analysed during the current study are available from the corresponding author on reasonable request.

## Appendix 1

**Table 2:** Some critical definitions of the notion “*talent*” in the business management literature

Author	Definition
Ashton and Morton (2005: 30)	*Talent can be identified as an employee who performs superior and possesses high-potential for further development.*
Avedon and Scholes (2010: 75)	*Talent alludes to significant individuals and groups with the key competencies that facilitate to reach short and long-term objectives of a company.*
Bagheri et al. (2020: 89)	*Talent refers to three distinct logic: a) capabilities and unique contributions of an individual; b) a significant employee; c) a definite and featured group in a company.*
Beechler and Woodward (2009: 274); McDonnell et al. (2011:178)	*Talent is the total output of an employee’s KSAs, capabilities and his/her ability to learn and develop.*
Bolander et al. (2017: 1533)	*Talent can be alluded as a strain of qualifications that are innate, but also must be formalized by the company to be fully operational and beneficial..*
Bonneton et al. (2019: 4)	*Talent can be defined as the employees anticipated to create the difference to business performance by their current and potential contribution.*
Buckingham and Vosburgh (2001: 21)	*Talent should reflect an employee’s iterative model of notion, perspective, or action that can be efficiently performed.*
Chabault et al. (2012: 328)	*Talent can be seen as a rare and distinguished asset specifically incorporated within an employee which facilitates his/her company in the way of competitive advantage.*
Crane and Hartwell (2019: 83)	*Talent is the combination of an employee’s human capital and social capital.*
Ewerlin and Süß (2016: 143); Thunnissen et al. (2013a: 1752)	*Both practitioners and academics generally identify talent as a combination of personal attributions which differentiates according to the type of task, the dynamics of the company and across time at the meantime, talent is usually attributed to the high performer and high potential employees.*
Hartmann et al. (2010: 176); Lewis and Heckman (2006: 141)	*Talent is identified as an unalterable employee’s knowledge, skill and abilities (KSAs), motivation, and also value-added contributions to the company.*
Iles et al. (2010: 180)	*Talent is a collection of distinct KSAs, like; leadership ability, emotional intelligence, analytical thinking, communication skills, ability to attract and inspire, operational skills, and result oriented practicalness* etc.
Jooss et al. (2019: 3); Swailes (2013: 33–34)	*Talent alludes to the employees who are currently or potentially contribute extra to firm performance.*
Meyers and van Woerkom (2014: 196); Silzer and Church (2009: 379)	*Talent is clarified as a potential, intended that it symbolizes the probability that employees can transform into something better than what they currently are.*
Kravariti and Johnston (2020: 81).	*Talent is delineated as an employee’s qualifications which leads him/her to outperform other employees.*
Latukha (2015: 1055); 171); Latukha (2018: 71)	*Talent is a natural gift, competence and capability which is sustained, rare and difficultly transferrable.*
Hedayati-Mehdiabadi and Li (2016: 6); Nilsson and Ellström (2012: 29)	*Talent is a reflection of an employee’s enduring capabilities which contributes to organizational performance.*
Nijs et al. (2014: 182)	*Talent refers to the systematically developed inborn competencies of employees that are assigned in tasks they enjoy, find interesting, and in which they eager to invest energy.*
Poocharoen and Lee (2013: 1187)	*Talent is the combination of competence, commitment, and contribution of an employee; competence refers to the ability to do, commitment refers to the devoting his/herself to do, and contribution refers to the value created by the employee.*
Schiemann (2014: 287)	*Talent is the congregate KSAs, values, actions and experiences of all employees combined to lead reaching business objectives.*
Tansley (2011: 268)	*Talent is an inborn capableness that demonstrates itself in a specific occupation or area and is attached to superior performance in a way.*
Thunnissen and Buttiens (2017: 408)	*Talent is the mixture of doing the work you like and doing it good.*
Tlaiss et al. (2017: 428)	*Talent can be referred as a potential which can be developed through learning and training.*

## Appendix 2

**Table 3:** Some important definitions regarding to the “*talent management”* concept in the business management literature

Author	Definition
Lewis and Heckman (2006: 140); Cui et al. (2018: 11)	*TM defined as planning and preparing future projections about employment requirements, and focusing on high-performing, high-potential talent.*
Barron (2008: 730); Bagheri et al. (2020: 88)	*Talent management (TM) refers to the procedure of identifying and developing talented employees through phases of interviewing, hiring, orienting and completely integrating them into the organization’s culture.*
Collings and Mellahi (2009: 304)	*Talent management can be defined as processes and activities that significantly contribute the company’s sustainable competitive advantage, that involve identifying key positions, developing and maintaining a talent pool to fill these positions and generating a differentiated HR architecture to facilitate filling these.*
Hausknecht et al. (2009: 270); Silzer and Dowell 2010: 18	*TM is the execution of collective strategies or systems developed to enhance firm productivity by developing superior processes for attracting, developing, utilizing and retaining employees with the necessary skills and aptitude to meet current and future business needs.*
Iles et al. (2010: 181)	*TM is the way of strategically managing the flow of talent along a company at aiming to match the right people with the right jobs at the right time based on strategic business objectives.*
Burbach and Royle (2010: 415)	*Talent management is a type of personnel management. It focuses on the knowledge, skill and abilities (KSAs) of the employee and on his/her potential for advancement to senior management roles by contributing the business success.*
Scullion et al. (2010: 106); Ewerlin and Süß (2016: 144)	*Talent management can be identified as the combined tasks of identifying, selecting, developing, appraising, motivating and retaining talent in order to enable and maintain the sustainable competitive advantage of the company.*
Tarique and Schuler (2010: 124); Cerdin and Brewster (2014: 248)	*Talent management is related to systematically operating HRM activities to attract, develop, and retain employees with high levels of human and social capital consistent with the strategic directions of the company in a dynamic, highly competitive environment.*
Bethke-Langenegger et al. (2011: 530)	*Talent management is a process which can be utilized to lead employees’ behaviour in a way that fits business needs.*
Schuler et al. (2011: 507)	*Talent management refers to the systematic utilization of significant policies and practices to handle the several talent challenges that a firm confronts.*
Raman et al. (2013: 336)	*TM could be referred as the contemplated and organized actions by firms to ideally select, develop, deploy and retain competent and committed talented employees for key positions which bear significant influences on the overall performance and competitive advantage of the organization.*
Gelens et al. (2013: 342)	*TM is the differential management of employees according to their relative potential to contribute to an organization’s competitive advantage.*
Cappelli and Keller (2014: 307)	*Talent management is the process which companies anticipate and encounter their demand for talent in strategic positions.*
Cascio and Boudreau (2016: 111)	*Talent management could be identified as the management and development of high-performing and high-potential employees in critical and important organizational roles.*
Festing and Schäfer (2014: 263)	*TM can be identified as a collection of all policies, practices, and systems that effect, bearing, and performance of talents, aiming at attraction and retention of talented employees.*
Al Ariss et al. (2014: 173-174); (Bonneton et al. (2019: 4)	*TM can be defined as a bunch of HR practices proposed to support the developing of a company’s talent pipeline for key and strategic positions.*
Crowley-Henry and Al Ariss (2018: 2059)	*TM can be defined as a way of identifying, selecting, recruiting, developing, and retaining talents to meet strategic goals of companies.*

## Appendix 3

**Table 4:** Profile of the participants

Type of Company	Occupation	Gender	Age	Years of Experience within the Industry	Education	Interviewee Code
Overseas-based Multinational Company	HR Manager	Male	41 Years and Over	11–15 Years	Bachelor’s Degree	P1
	HR Specialist	Male	20–30 Years	1–5 Years	Bachelor’s Degree	P2
	HR Specialist	Female	20–30 Years	6–10 Years	Bachelor’s Degree	P3
Overseas-based Multinational Company	HR Manager	Male	41 Years and Over	21 Years and Over	Master’s Degree	P4
	HR Assistant Manager	Female	41 Years and Over	21 Years and Over	Bachelor’s Degree	P5
	HR Specialist	Female	20–30 Years	1–5 Years	Master’s Degree	P6
Overseas-based Multinational Company	HR Assistant Manager	Female	41 Years and Over	21 Years and Over	Bachelor’s Degree	P7
	HR Chief	Male	20–30 Years	6–10 Years	Master’s Degree	P8
	HR Specialist	Female	20–30 Years	1–5 Years	Master’s Degree	P9
Turkish Large-scale Company	HR Assistant Manager	Male	31–40 Years	16–20 Years	Bachelor’s Degree	P10
Turkish Medium-sized Enterprise	HR Manager	Female	31–40 Years	6–10 Years	Bachelor’s Degree	P11
Turkish Multinational Company	HR Specialist	Female	20–30 Years	6–10 Years	Master’s Degree	P12
Turkish Multinational Company	HR Specialist	Female	20–30 Years	1–5 Years	Master’s Degree	P13
Turkish Large-scale Company	HR Business Partner	Female	31–40 Years	6–10 Years	Master’s Degree	P14
Turkish Multinational Company	HR Specialist	Female	31–40 Years	6–10 Years	Master’s Degree	P15
Turkish Large-scale Company	HR Specialist	Male	31–40 Years	1–5 Years	Bachelor’s Degree	P16
Turkish Multinational Company	HR Specialist	Male	31–40 Years	6–10 Years	Bachelor’s Degree	P17
Turkish Large-scale Company	HR Business Partner	Male	31–40 Years	11–15 Years	Master’s Degree	P18
Turkish Multinational Company	HR Manager	Female	31–40 Years	6–10 Years	Bachelor’s Degree	P19
Turkish Multinational Company	HR Manager	Female	31–40 Years	11–15 Years	Master’s Degree	P20
Turkish Multinational Company	HR Specialist	Female	31–40 Years	1–5 Years	Master’s Degree	P21
Joint Venture (Turkish-Foreign)	HR Manager	Male	41 Years and Over	21 Years and Over	Bachelor’s Degree	P22
Turkish Multinational Company	HR Specialist	Female	31–40 Years	1–5 Years	Bachelor’s Degree	P23
Turkish Multinational Company	HR Manager	Female	20–30 Years	1–5 Years	Bachelor’s Degree	P24
Turkish Multinational Company	HR Manager	Female	41 Years and Over	21 Years and Over	Master’s Degree	P25
Turkish Large Scale Company	HR Manager	Female	31–40 Years	16–20 Years	Bachelor’s Degree	P26
Joint Venture (Turkish-Foreign)	HR Business Partner	Female	31–40 Years	1–5 Years	Master’s Degree	P27
